# Indication of Premelanosome Protein (PMEL) Expression Outside of Pigmented Bovine Skin Suggests Functions Beyond Eumelanogenesis

**DOI:** 10.3390/genes11070788

**Published:** 2020-07-13

**Authors:** Jacqueline Knaust, Rosemarie Weikard, Elke Albrecht, Ronald M. Brunner, Juliane Günther, Christa Kühn

**Affiliations:** 1 Institute for Genome Biology, Leibniz Institute for Farm Animal Biology (FBN), 18196 Dummerstorf, Germany; jacqueline_knaust@yahoo.de (J.K.); weikard@fbn-dummerstorf.de (R.W.); brunner@fbn-dummerstorf.de (R.M.B.); guenther@fbn-dummerstorf.de (J.G.); 2 Institute for Muscle Biology and Growth, Leibniz Institute for Farm Animal Biology (FBN), 18196 Dummerstorf, Germany; albrecht@fbn-dummerstorf.de; 3 Faculty of Agricultural and Environmental Sciences, University Rostock, 18059 Rostock, Germany

**Keywords:** PMEL, *Bos taurus*, expression, skin, pigmentation

## Abstract

The premelanosome protein (PMEL) is important for fibril formation within melanosomes during vertebrate melanogenesis. Fibrils form a matrix for pigment deposition within pigmented tissues such as skin and hair. PMEL mutations are known to modulate eumelanic pigmentation in vertebrates. However, in bovines, PMEL mutations were also found to alter pheomelanic pigmentation resulting in coat color dilution. Furthermore, epistatic effects of a mutated PMEL allele were detected in the phenotypic expression of the bovine hair defect “rat-tail syndrome” (RTS) characterized by charcoal coat color and hair deformation. Reports about *PMEL* gene expression in non-pigmented tissues raised the hypothesis that there may be unknown functions of the PMEL protein beyond eumelanin deposition to PMEL fibrils. In our study, we analysed the PMEL protein expression in pigmented skin and non-pigmented bovine tissues (non-pigmented skin, thyroid gland, rumen, liver, kidney, and adrenal gland cortex). We found that a processed form of the bovine PMEL protein is expressed in pigmented as well as in non-pigmented tissues, which is in line with gene expression data from targeted RT-PCR and whole transcriptome RNAseq analysis. The PMEL protein is located in membranes and within the cytosol of epithelial cells. Based on our data from bovine tissues, we concluded that at least in cattle PMEL potentially has additional, yet unexplored functions, which might contribute to effects of PMEL mutations on pheomelanin coat color dilution and charcoal coat color in RTS animals. However, indication of PMEL protein in unpigmented cells and tissues will require further confirmation in the future, because there have been no confirmed reports before, which had detected bovine PMEL protein with specific antibodies either in pigmented or unpigmented tissue.

## 1. Introduction

The silver homolog premelanosome protein (PMEL, alternative names: Pmel-17, SILV, gp100 and ME20) has an important function in pigmentation of vertebrates. It is known that the protein is responsible for fibril formation within eumelanosomes [1], and it determines the elliptical eumelanosomal shape [2]. In contrast, pheomelanosomes show no fibril formation presumably due to the lack of PMEL expression [3]. The fibrillary structures laterally assemble into sheets, which serve as a matrix for eumelanin deposition (reviewed by Watt and co-workers [4]). Mutations in the *PMEL* gene are associated with coat color dilution in the mouse [5], chicken [6], dog [7] and horse [8]. In cattle, PMEL function seems to be unique because effects of genetic variants are not restricted to eumelanic coat color dilution as reported for other vertebrates. The non-synonymous mutation *PMEL c.64G>A* (NM_001080215.2) in the signal peptide region of the bovine PMEL protein (NP_001073684) is causative for the *Dilution* locus (https://omia.org/OMIA001545/9913/), associated with extreme coat color dilution in the Charolais cattle breed [9]. A three-base-pair deletion c.50_52delTTC also in the N-terminal region of the bovine PMEL protein is responsible for coat color dilution in Highland and Galloway breeds [10]. These mutations in the bovine *PMEL* gene affect eumelanic as well as pheomelanic pigmentation [9,10,11]. This is remarkable because in other species pheomelanosomes are assumed to lack PMEL expression [3]. Furthermore, the PMEL locus has been identified as one component of a complex interaction of three loci underlying the genetic defect “rat tail syndrome” (RTS) in cattle (https://omia.org/OMIA001544/9913/) [12]. RTS is exclusively expressed in animals with a eumelanic background. This genetic defect was observed in calves produced by crossing animals from German Holstein with animals from the Charolais breed. The calves have short, curly, sometimes sparse hair, and a lack of normal tail hair development. Besides substantial effects on the hair structure, this defect is also associated with genetically-determined variation in coat color; animals with the RTS phenotype exhibit a darker grey (charcoal) coat color than animals without RTS phenotype. Whether this is due to direct effects on melanocytes or whether PMEL in cattle might have additional functions beyond eumelanocytogenesis, which contribute to the coat color and hair formation variation in RTS animals, is under debate. Thus, the full role of PMEL effects beyond eumelanogenesis remains to be determined. A prerequisite for proving additional PMEL functions is the verification of its expression also outside of eumelanocytes. However, there is controversy regarding pigment cell-specific PMEL expression. Recently, a comprehensive multi-tissue survey in humans showed indication of PMEL protein expression in further tissues additional to melanocytes (www.proteinatlas.org). Although PMEL has been widely used as melanoma tumour marker [13], and there are other reports indicating that PMEL protein expression is restricted to pigment cells [14], Kuehn and Weikard (2007b) have detected bovine *PMEL* mRNA gene expression in pigmented and non-pigmented tissues and also identified different *PMEL* transcripts generated by alternative splicing [15]. Based on the indication of *PMEL* expression outside the eumelanocyte lineage, it has been hypothesized that the PMEL protein has functions beyond eumelanosomes that are still unknown. Consequently, in this study we monitored potential PMEL protein expression in different pigmented and non-pigmented tissues including details in hair structure and obtained indication that there are PMEL-expressing cells outside the eumelanocyte lineage.

## 2. Materials and Methods 

### 2.1. Ethics Statement

All applicable international, national, and/or institutional guidelines for the care and use of animals were followed. All experimental procedures were carried out in compliance with German animal welfare guidelines and were monitored by the competent authorities of the State Office for Agriculture, Food Safety and Fisheries, Mecklenburg-Vorpommern, Germany. The protocols were approved by the Animal Protection Board of the Leibniz Institute for Farm Animal Biology as well as by the Animal Care Committee of the State Mecklenburg-Western Pomerania, Germany on 27 March 2003 (State Office for Agriculture, Food Safety and Fishery; LALLF M-V/ Rostock, Germany, TSD/7221.3-2.1-010/03).

### 2.2. Animals and Sampling

Tissue samples for Western blot analyses were taken immediately after slaughter from two adult eumelanic F_2_ black and white piebald individuals (male and female) of an experimental cattle-resource-cross population generated from German Holstein and Charolais. Both animals had the eumelanic genotype *E^D^/e* at the *melanocortin 1 receptor (MC1R)* [16] and the non-dilute wild type *dc+/dc+* genotype at the *PMEL c.64G>A* locus [9]. Samples were collected from pigmented (eumelanic black) and non-pigmented (white) skin (prescapular region, immediately adjacent sections of pigmented, and non-pigmented skin), liver, kidney, adrenal gland (cortex), thyroid gland, and rumen. For immunohistochemistry (IHC), samples from pigmented and non-pigmented skin, rumen, and thyroid gland were investigated. The hair of all skin samples was removed with a scalpel, and all samples were cut and frozen in liquid nitrogen and stored at −80 °C until further use. For Western blot, the tissues were dissected in grain-shaped pieces, and for IHC studies, 1 cm^3^ tissue cubes were prepared. 

### 2.3. Protein Isolation

Standard procedures as described previously [17] and modified according to Leonhardt and co-workers were used for protein extraction from tissue samples [18]. Samples of pigmented and non-pigmented skin, liver, kidney, adrenal gland (cortex), thyroid gland, and rumen were ground with liquid nitrogen. In a first extraction step, 1 g of these ground samples were incubated overnight at 4 °C with 1.5 ml RIPA buffer (25 mM Tris-HCI pH 7.6, 150 mM NaCl, 1% NP40, 1% Na-deoxycholate, 0.1% SDS, 1 mM EDTA, 8 M urea). High concentrations of urea were used as described [17] to increase the solubility of proteins from cornified tissues. After centrifugation (15,000× g, 10 min, 4 °C), the supernatants with the highly-soluble protein fractions were collected, and protein concentrations were determined by a Bradford assay using Roti-Quant® (Carl Roth, Karlsruhe, Germany) according to the manufacturer’s instructions. The pellets containing the lowly-soluble protein fractions were used for a second, more rigorous extraction step according to Leonhardt and co-workers [18]. Briefly, pellets were incubated for 10 min in 500 µL of lysis buffer (PBS, 1% SDS, 1% 2-mercaptoethanol) at room temperature, then for 10 min at 100 °C. Protein concentration was measured using a Bradford assay. All samples were denatured for 5 min at 95 °C with Laemmli sample buffer, separated on 10% polyacrylamide gels and stained with Coomassie® Brilliant Blue R-250 (Serva, Heidelberg, Germany) for quality control of the protein extracts.

Recently, there was proof of PMEL protein expression in the human hepatoma cell line HEPG2 [19]. Thus, whole cell lysates from the HEPG2 cell line were used as positive control for primary anti-(human) PMEL antibody binding. To this end, HEPG2 (ATCC HB-8065, ATCC, Wesel, Germany) cells were cultured in Eagle’s Minimum Essential Medium (EMEM). Confluent cells were washed twice in PBS solution and lysed in Laemmli sample buffer while boiling for 5 min. Human fibroblast lysates (FHs 173We, Santa Cruz Biotechnology, Inc., Dallas, TX, USA) were included as negative control, because Hellström and co-workers had demonstrated lack of PMEL protein expression in these cells [2]. As *PMEL* transcript analysis in a panel of bovine tissues identified *PMEL* expression in all tissues tested [15], no confirmed bovine negative control was available. MAC-T cells from a bovine mammary epithelial cell line [20] that were cultivated in DMEM (Lonza) supplemented with 10% FCS (PAN Biotech) on polystyrene tissue culture plates (CELLSTAR, Greiner Bio-One, Frickenhausen, Germany) served as proof of endogenous cellular PMEL expression. Lysate protein was isolated from these cells as described above.

### 2.4. Western Blot

An antibody directed against the N-terminus of the human PMEL protein was used for detection of the orthologous bovine PMEL protein in various bovine tissues. The polyclonal rabbit anti-(human) PMEL antibody (anti-PMEL, ARP46802_T100, Aviva Systems Biology, Biozol Diagnostics, Eching, Germany) is directed against the peptide sequence HFLRNQPLTFALQLHDPSGYLAEADLSYTWDFGDSSGTLISRALVVTHTY of human PMEL aa 231–280 (Accession: NP_001186983.1), which has 93% sequence homology to aa 231–280 of the bovine PMEL protein (Accession: NP_001073684.2). The epitope recognized by the antibody comprises a region that could be found in the full length P1 and P2 forms of PMEL as well as in Mα (aa 26–469) and MαC fragments (aa 235–469) [21,22]. Twenty µg of total lysate protein from the highly and lowly-soluble protein fraction of pigmented and non-pigmented tissues, lysates from HEPG2 cells (positive control), human fibroblasts (negative control), and MAC-T cells (proof of endogenous cellular PMEL expression), respectively, were separated on 10% polyacrylamide gels. The gels were transferred to nitrocellulose membranes (Protran, 0.45 µm, Whatman^TM^, Sigma-Aldrich, Taufkirchen, Germany) by electro-tankblotting (Bio-Rad, München, Germany). Following transfer, nonspecific antibody binding was blocked by 5% bovine serum albumin (BSA, Albumin Fraction V, pH 5.2, Carl Roth, Karlsruhe, Germany). The blocking solution was generated with PBS solution containing the detergent Tween^®^ 20 (0.1%). Primary antibody incubation was performed overnight at 4 °C with the anti-PMEL IgG (1.25 µg/ml final concentration). Subsequently, the membranes were incubated for one hour with horseradish peroxidase-conjugated goat anti-rabbit IgG (1:10,000, #7074, Cell Signaling Technology, Inc., New England Biolabs, Frankfurt, Germany). Protein signals were obtained using a chemiluminescence substrate. Briefly, 1 ml of 1.4 mM luminol (Sigma-Aldrich, Taufkirchen, Germany) diluted in 100 mM Tris/HCl, pH 8.6 were mixed with 0.3 µL 30% H_2_O_2_ and 100 µL 33.5 µM para-hydroxycoumaric acid (Sigma Aldrich, Taufkirchen, Germany) diluted in DMSO. Protein bands were detected using the ChemiDoc^TM^ MP Imaging System and visualized using Image Lab^TM^ Software (Bio-Rad). PMEL protein intensities were compared to alpha tubulin levels. To this end, tubulin was detected on the stripped membrane (Restore^TM^ PLUS Western Blot Stripping Buffer, Life Technologies) using anti-alpha tubulin (11H10) rabbit monoclonal primary antibody (1:1000, #2125, Cell Signaling Technology, Inc.) and the horseradish peroxidase-conjugated goat anti-rabbit IgG secondary antibody (1:10,000, #7074, Cell Signaling Technology, Inc.). 

### 2.5. Immunohistochemistry (IHC)

To reveal the specific cellular origin of the PMEL protein expression signals from the Western blot analysis, we performed immunohistochemistry analyses in tissues that had displayed protein expression signals in Western blots. Cryosections of pigmented and non-pigmented skin (10 µm thickness), thyroid gland (10 µm), and rumen (8 µm) were generated using a Leica CM3050 S cryostat microtome (Leica, Bensheim, Germany). After the sections had been air dried, fixed with ice-cold acetone, and washed with PBS, unspecific binding of the secondary antibody was blocked for 15 min with PBS including 10% goat serum. Subsequently, sections were incubated overnight with anti-PMEL (1:100) primary antibody at 4 °C in a humidity chamber. Specific binding of primary antibody was detected with the goat anti-rabbit IgG secondary antibody conjugated with Alexa Fluor® 488 (#A11034, Life Technologies, Darmstadt, Germany). A counterstaining of the nuclei was performed using 1 μg/ml Hoechst 33258 (Sigma-Aldrich). After staining, the slides were covered with ProLong Diamond Antifade Mountant (Fisher Scientific, Schwerte, Germany) and appropriate cover-slips. Sudan black B (0.1% in 70% ethanol) was used for reducing intense auto fluorescence in affected tissues. The same protocol, except for incubation with the primary antibody, was used for the negative controls. Immunofluorescence was visualized using a Nikon Microphot SA fluorescence microscope (Nikon, Duesseldorf, Germany), a CC-12 high resolution color camera (OSIS, Muenster, Germany), and an image analysis system equipped with CELL^F software.

### 2.6. Immunocytochemistry

For immunocytochemistry, MAC-T cells were seeded on coverslips. After overnight culture growth, coverslips were washed three times with PBS and fixed with ice-cold methanol (10 min, −20 °C). Anti-PMEL (1:100) primary antibody was incubated overnight at 4 °C. Unspecific antibody binding was blocked with Roti-Immunoblock (Carl Roth GmbH). Goat anti-rabbit IgG secondary antibody conjugated with Alexa Fluor® 488 (1:1000; # A11034, Life Technologies, Germany) was used to detect specific binding of the primary antibody. Nuclei were counterstained with 600 nM DAPI (Carl Roth GmbH). The same protocol, excluding incubation with the primary antibody, was used for the negative controls. Cells were mounted with DABCO (Carl Roth GmbH) and analyzed with the Axio Observer.Z1 inverted fluorescence microscope (Carl Zeiss Microscopy GmbH) using the Plan-Apochromat 63x/1.4 Oil DICIII objective. 

## 3. Results

### 3.1. PMEL Signals Are Detected in Pigmented and Non-Pigmented Tissues

Whereas *PMEL* mRNA expression had been described in vertebrate non-pigmented tissues [15], final proof of respective PMEL protein expression was still under debate. During maturation, PMEL undergoes various processing events like proteolytic cleavages and glycosylations. Glycosylation of proteins may vary from one species to another and also between cancer and wild type cells. The widely-used diagnostic PMEL antibodies for human malignant melanoma like HMB45 are known to only recognize epitopes requiring specific glycans [23]. Others like HMB50 and NKI-beteb do not work well in Western blot applications [24]. 

Therefore, for our analyses we chose a peptide antibody against an amino acid sequence containing the PKD (polycystic kidney disease) domain region of the human PMEL with high sequence homology to the bovine PMEL protein. Within this PMEL region, there were no known glycosylation sites in the human or mouse protein. Based on this epitope we assumed that in cattle the antibody should be able to recognize the P1 precursor, the posttranslational glycosylated full-length P2 form, and the complete Mα cleavage product, as well as the further cleaved MαC (also known as Mα’) fragment [22]. The non-glycosylated P1 precursor is known to migrate with M_r_ of ~100 kDa [21]. The Golgi-glycosylated short-lived P2 form migrates in a reducing SDS-PAGE with M_r_ of ~120 kDa [21]. There are contradictory findings about the M_r_ of the cleavage product Mα. It ranges from ~80 kDa [25] to ~100 kDa [21]. The further cleaved MαC fragment migrates with M_r_ of ~35 kDa. For proof of PMEL protein expression in non-pigmented tissue by Western blotting, we needed to test, whether this antibody directed against a human PMEL peptide (anti-PMEL) specifically binds to bovine PMEL protein. To this end, the highly soluble protein fractions of pigmented skin from two piebald eumelanic adult cattle (male and female) and the human hepatoma cell (HEPG2) lysate served as positive controls and human fibroblast (FHs 173We) lysate as negative controls. HEPG2 cells had been demonstrated to express PMEL at protein level [19]. 

**Figure 1:**
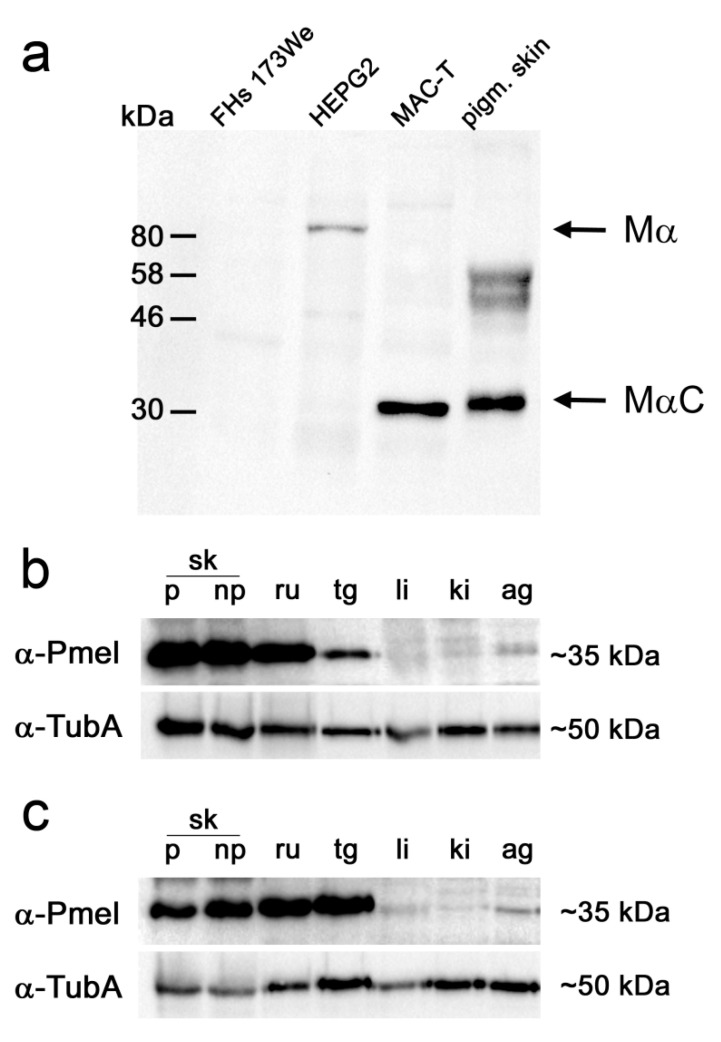
Western blot analyses indicating specific premelanosome (PMEL) protein detection using anti-PMEL antibody (α-PMEL). (**a**) Whole cell lysate of human fibroblasts (FHs 173We, negative control), human HEPG2 cells (positive control), MAC-T (bovine mammary epithelial cell line), and highly-soluble protein extract from pigmented skin (pigm. skin). Molecular weight is indicated on the left side. Mα and MαC are labelled as suggested fragments of the processed PMEL products. The two bands detected in pigmented skin (~56 and ~50 kDa) originate from unspecific signals from the secondary antibody. (**b**) Western blot analysis with the anti-PMEL antibody (α-PMEL) and anti-alpha tubulin (α-TubA) in the highly soluble protein fraction and (**c**) Western blot analysis with the anti-PMEL antibody (α-PMEL) and anti-alpha tubulin (α-TubA) in the lowly-soluble protein fraction of pigmented and non-pigmented tissues. Skin (sk), pigmented (p), non-pigmented (np), rumen (ru), thyroid gland (tg), liver (li), kidney (ki), and adrenal gland cortex (ag). On the right side, the molecular weight of the detected bands is indicated.

As expected, the HEPG2 lysate displayed a specific signal with the anti-PMEL antibody according to the length of ~80 kDa (Figure 1a). For the pigmented skin sample, we obtained specific PMEL signals at approximately 35 kDa (Figure 1a). No signal was obtained for the human fibroblast cell lysate, serving as negative control. After testing the specificity of the primary PMEL antibody, protein extracts originating from the highly- and lowly-soluble protein fractions of pigmented, and non-pigmented tissues were monitored in independent subsequent Western blot analyses. Tissues were selected due to *PMEL* mRNA expression as previously described [15]. Using the anti-PMEL antibody, a ~35 kDa band indicating the bovine PMEL was detected. Whereas liver, kidney, and adrenal gland cortex revealed weak signals in both protein fractions, pigmented and non-pigmented skin, rumen, and thyroid gland exhibited strong signals (Figure 1b,c). 

### 3.2. Bovine PMEL Protein Seems to Be Present not Only in Cells of the Melanocyte Lineage

**Figure 2:**
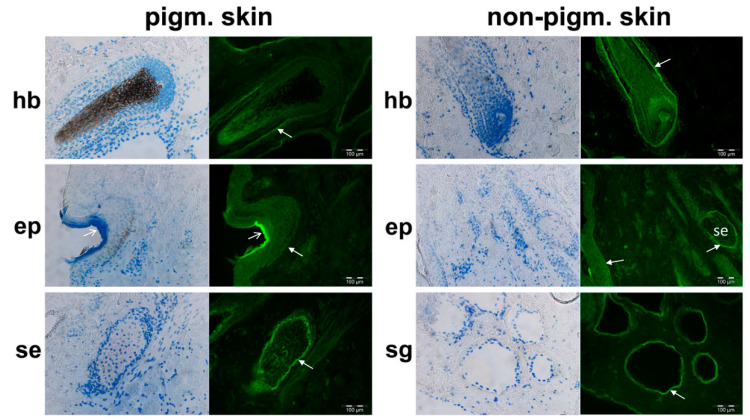
Immunofluorescence micrographs of different pigmented and non-pigmented skin for detection of the cellular location of the bovine PMEL protein with a rabbit anti-(human) PMEL antibody. (**hb**) Hair bulb (see dark brown melanin pigment granulae in hair of pigmented skin and missing melanin granulae in non-pigmented skin), arrows indicate the PMEL-positive root sheath cells; (**ep**) epidermis, arrows indicate PMEL-positive cells of stratum basale, open arrow indicates autofluorescence of stratum corneum; (**se**) sebaceous gland; and (**sg**) sweat gland, arrows indicate PMEL-positive epithelial cells. Left, bright field microscopy merged with image displaying nuclei stained with Hoechst 33258 (blue) and right, immunohistochemical fluorescence images of PMEL detection (green).

**Figure 3:**
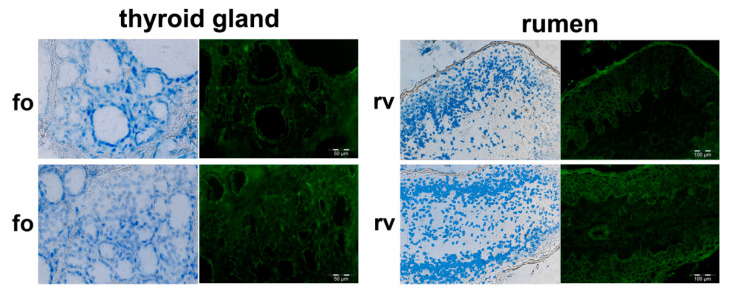
Immunofluorescence micrographs of different non-pigmented tissues for detection of the cellular location of the bovine PMEL protein with a rabbit anti-(human) PMEL antibody. (**fo**) Two regions of thyroid gland with thyroid gland follicles and (**rv**) two regions of rumen with rumen villi. Left, bright field microscopy merged with image displaying nuclei stained with Hoechst 33258 (blue) and right, immunohistochemical fluorescence images of PMEL detection (green).

Subsequent IHC analyses served to elucidate the specific cellular origin of the PMEL signals in the tissues that had been obtained in Western blot analyses. We observed fluorescence signals with the anti-PMEL antibody in melanocytes and also in cells not associated with pigmentation—in pigmented and in non-pigmented skin (Figure 2), in the thyroid gland, and in the rumen (Figure 3). In addition to the hair bulb and the hair shaft, PMEL signals in the pigmented and in non-pigmented skin arose from cells in the root sheaths of the hair follicles. Furthermore, indication of PMEL expression was obtained in epithelial cells of sweat glands, cells of the sebaceous gland, and in the stratum basale of the epidermis. In general, we obtained no data suggesting a reduced PMEL protein expression in non-pigmented skin compared to pigmented skin (Figure 1b,c and Figure 2). Negative controls, omitting the primary antibody, showed very weak unspecific binding of the secondary antibody, but some autofluorescence, e.g., probably in elastin fibres, in the hair shaft and in the stratum corneum, and with different patterns than the specific signals (see Supplemental Figure S1). 

In the thyroid gland, our studies revealed signals indicating PMEL expression in the epithelial cells of the follicles (Figure 3). The PMEL signals in rumen were detectable in cells of the stratum basale as well as in the stratum spinosum and the stratum corneum of the villi (Figure 3), whereas the signals in the stratum corneum of the villi were identified as autofluorescence.

### 3.3. Indication of Putative Bovine PMEL Protein Expression in non-Melanocyte Cells

Due to the “net-like” structure of the fluorescence signals from some cells in the hair follicle (e.g., root sheath, see Figure 2), cells of the sebaceous gland (see Figure 2), and the follicular cells of the thyroid gland (Figure 3), there is indication that PMEL is localized in the cell membranes of epithelial cells. In contrast, in the hair bulb, in skin epidermal cells (Figure 2), and in the rumen (Figure 3), diffuse cellular signals were observed (e.g., within the epithelial layer of the rumen villi), which provided support for a cytosolic location of the PMEL protein product detectable with the anti-PMEL antibody.

**Figure 4:**
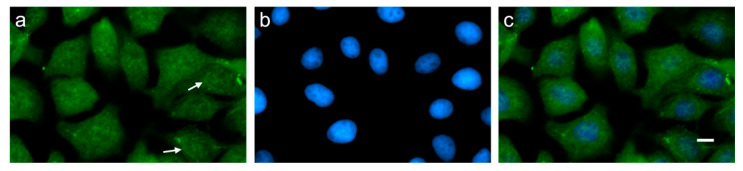
Immunofluorescence micrographs of mammary epithelial MAC-T cells, detecting subcellular localization of the bovine PMEL protein with a rabbit anti-(human) PMEL antibody. (**a**) Immunocytochemical fluorescence images of PMEL detection (green), (**b**) nuclei stained with DAPI (blue), and (**c**) merged images. Note PMEL protein displays a granular distribution pattern throughout the cytoplasm with perinuclear enrichment (arrows). Scale bar 10 µm.

Our results on the protein level confirm previous reports about *PMEL* mRNA expression in bovine [9] or other mammalian (e.g., [26] or Single Cell Expression Atlas (https://www.ebi.ac.uk/gxa/sc/home)) non-pigmented tissues [15], which do not have any function related to pigmentation (non-pigmented skin, rumen, liver, kidney, thyroid gland, and adrenal gland cortex). In addition, in pigmented skin, we also found indication of putative PMEL protein expression in sweat gland and sebaceous gland cells, although those cells do not belong to the melanocyte lineage and are not assumed to incorporate melanosomal products. Furthermore, our IHC data excluded that PMEL expression signals detected by Western blot analysis were due to dispersed mature melanocytes, migrating melanocyte stem cells, or mesenchymal stem cells differentiating into melanocytes in non-pigmented tissues. To exclude the possibility that the PMEL protein signal detected in non-melanocytes is acquired from external PMEL RNA and/or protein sources as described for exosomal transfer [27], we investigated an in vitro culture of a bovine mammary epithelial cell line for PMEL protein expression. Western blot analysis (Figure 1a) as well as immunofluorescence microscopy (Figure 4) confirmed that these epithelial cells in isolated cell culture also displayed a PMEL signal suggesting that the mammary epithelial cells might express the PMEL protein endogenously.

## 4. Discussion

The premelanosome protein PMEL is known for its role during pigment formation and eumelanosome development and in human medicine it is even used as melanoma tumour marker due to its assumed melanocyte-specific expression at protein level [13,14]. However, it still remains an open question, as to what the specific PMEL functions in pheomelanosomes are and whether this protein has other roles beyond fibril formation in melanocytes. Data from previous literature [19] suggest PMEL protein expression in other cell types in addition to melanocytes, e.g., in the human hepatocarcinoma cell line HEPG2. In the HEPG2 cell line, we observed a PMEL signal migrating with M_r_ of ~80 kDa. This may represent the Mα fragment. For the bovine tissues investigated, our results are in line with previous reports about *PMEL* mRNA expression [15]. Furthermore, our data provide indication that processed PMEL protein might be expressed in vertebrate non-pigmented tissues, which do not have any function related to pigmentation and are known to be devoid of melanocytes (non-pigmented skin, rumen, liver, kidney, thyroid gland, and adrenal gland cortex). In pigmented skin, we also found indication of putative PMEL protein expression in sweat glands and sebaceous gland cells, although these cells do not belong to the melanocyte lineage, and it is not assumed that they incorporate melanosomal products. PMEL expression outside of pigmented tissues had also been described by Yajima and Larue (2008) [26] in sections of the murine heart, which the authors could assign to melanocytes dispersed in this organ. We cannot formally exclude that some of the signals obtained in our Western blot and IHC analyses in non-pigmented tissues were generated by dispersed melanocytes. However, our IHC results provide some indication supporting a cell type origin of the PMEL protein outside of melanocytes in the MAC-T cell line and in pigmented skin and non-pigmented tissues. One observation indicating that indeed non-melanocytes could be capable of synthesizing PMEL protein in *Bos taurus*, is our data suggesting that PMEL protein is expressed in in vitro culture of MAC-T mammary gland epithelial cells. Another indication of PMEL expression was obtained for tissue-specific cells in the thyroid gland or rumen by Western blot analysis, which excludes that the observed PMEL expression might be due to dispersed mature melanocytes, migrating melanocyte stem cells, or mesenchymal stem cells differentiating into melanocytes in non-pigmented tissues. Rather, the PMEL-specific IHC signals seem to originate from genuine tissue-intrinsic cells, which express PMEL on the protein level. PMEL expression in non-pigmented tissues would be particularly striking because this protein is considered a very relevant pigment cell-specific marker. However, our results are in line with recent data that report that, in humans, PMEL protein expression could also be detected in non-pigmented tissues, e.g., in the kidney or adrenal gland, in addition to the expression in skin [28]. The thyroid gland and liver had not shown PMEL protein expression in human, although RNA sequencing data had provided FPKM values for *PMEL* mRNA expression exceeding 1 in several samples [28], which suggests that some *PMEL* transcripts should also be present in this tissue. Latest results obtained from the Single Cell Expression Atlas data base (https://www.ebi.ac.uk/gxa/sc/home) indicate that PMEL expression has indeed been observed in a variety of cell types in other species (e.g., human pancreatic cells, kidney cells, and testis as well as in cells from murine small intestine or heart valve leaflet). This is in line with own previous data. In a targeted RT-PCR analysis, we were able to demonstrate PMEL transcripts in a variety of tissues [15]. Analogous results were obtained in recent whole transcriptome RNAseq experiments of liver and rumen, which confirmed PMEL expression in those tissues ([29], see deposited files in the Functional Annotation of Animal Genomes (FAANG) database (https://data.faang.org/dataset) under project number PRJEB3457).

Even though no specific quantitative analysis was performed, our data from Western blot images did not provide any indication of a difference in expression levels of the 35 kDa PMEL fragment between pigmented and non-pigmented skin. This is particularly noteworthy because at transcript level *PMEL* mRNA expression showed strong differences between pigmented and non-pigmented skin [15]. Donatien and Orlow [30] had described an increase in PMEL immunoreactivity upon decrease of pigmentation, and Raposo and Marks had described a complete masking of PMEL fibrils through melanin molecules at the end of processing in stage IV melanosomes [31]. Thus, the phenomenon of seemingly elevated PMEL protein expression in non-pigmented skin determined by immunological detection methods might be explained by a “masking” of PMEL by eumelanin in mature melanosomes stage II–IV in pigmented skin. 

It seems that PMEL may have functions in epithelial cells of pigmented and non-pigmented tissues in addition to melanin-associated fibril formation in melanocytes. Melanocytes are present only in pigmented tissues. Recently, sweat glands were reported to be a niche for melanocyte precursor cells [32], which was concluded from very punctual PMEL expression in the neonatal secretory portion of sweat glands. Thus, it cannot be excluded that the PMEL protein signals in sweat glands originate from such melanocyte precursor cells. Other non-classical melanocytes with unexpected localisation in the inner ear, meninges, bones, and heart of mice have been described in the literature [26,33,34,35,36]. Classical melanocytes are described as taking the dorso-lateral pathway during development from melanoblasts to melanocytes [26,37]. However, non-pigmented skin in adult piebald cattle, and the other non-pigmented tissues, that showed indication of PMEL protein expression in our study (e.g., thyroid gland or rumen), are commonly accepted to be devoid of melanocytes. Thus, the detected signals in Western blot analyses should be due to endogenous PMEL expression from keratinocytes in skin and epithelial cells in the other non-pigmented tissues, for which we obtained indication by IHC analysis. In pigmented skin, expression of bovine PMEL protein was not restricted to melanocytes as demonstrated by PMEL protein signals in keratinocytes, which is probably due to the concurrent transfer of eumelanin and PMEL between melanocytes and keratinocytes [38,39] via membrane-bound melanosomes [40]. This is in contrast to the unexpected PMEL signals e.g., in sebaceous glands, because currently no transfer of melanins into these cells has been described. 

The bovine PMEL protein seems to differ from the PMEL protein in other mammalian species in terms of additional functions, as has already been demonstrated with respect to pigmentation. Functional analysis in mice had shown an absence of PMEL expression in pheomelanin-producing melanocytes [3]. This corresponds to the observation that mutations in the *PMEL* gene affect only eumelanic coat color dilution as described for many species [6,7,8]. In contrast, a mutated bovine PMEL protein affects not only eumelanin-producing melanocytes but also pheomelanocytes [9,11]. Thus, it can be assumed that the bovine PMEL could undergo another processing and might therefore have a different secondary and tertiary structure than that of other species. This hypothesis might explain differences in PMEL detection compared to other species. To our knowledge, there have been no confirmed reports before, which have detected bovine PMEL protein with specific antibodies either in pigmented or unpigmented tissue. However, we point out that the indication of PMEL protein expression in unpigmented cells and tissues will require confirmation in future studies due to the antibody applied, which has not yet been comprehensively characterized.

Data from our earlier study in cattle [12] have suggested that PMEL might play a role in hair formation and hair color additional to eumelanin dilution as indicated by epistatic effects of a mutated PMEL allele on the phenotypic expression of the bovine hair defect “rat-tail syndrome” (RTS). RTS is a bovine form of hypotrichosis restricted to pigmented hair coat generating differences in hair color, structure, and length between pigmented and non-pigmented areas in piebald individuals [41]. Thus, PMEL contributes not only to hair pigmentation, but may also be related to hair formation. However, the specific molecular and cellular mechanisms underlying the genetic RTS defect are still unknown. To our knowledge, PMEL had not been associated with hair formation or structure in any other mammalian species. The relationship between RTS and potential PMEL protein functions, beyond eumelanin pigmentation, needs to be investigated in more detail in further studies.

## Supplementary Materials

The following are available online at https://www.mdpi.com/2073-4425/11/7/788/s1. Supplemental Figure S1. Immunofluorescence-micrograph of negative control images showing autofluorescence and minor unspecific binding of the secondary Alexa Fluor 488 goat anti-rabbit IgG antibody.
