# Supplementary material for: Indication of Premelanosome Protein (PMEL) Expression Outside of Pigmented Bovine Skin Suggests Functions Beyond Eumelanogenesis
Source: Genes (Basel). 2020 Jul 13;11(7):788. doi: 10.3390/genes11070788 (PMC7397160; doi:10.3390/genes11070788)

Supplemental Figure 1

Immunofluorescence-micrograph: Negative control images showing autofluorescence and minor unspecific bindings of the secondary Alexa Fluor 488 goat anti-rabbit IgG antibody (**Right**) in hair bulb (hb), epidermis (ep), thyroid gland (tg), and rumen (ru). **Left**: Bright field microscopy merged with image displaying nuclei stained with Hoechst 33258 (blue).

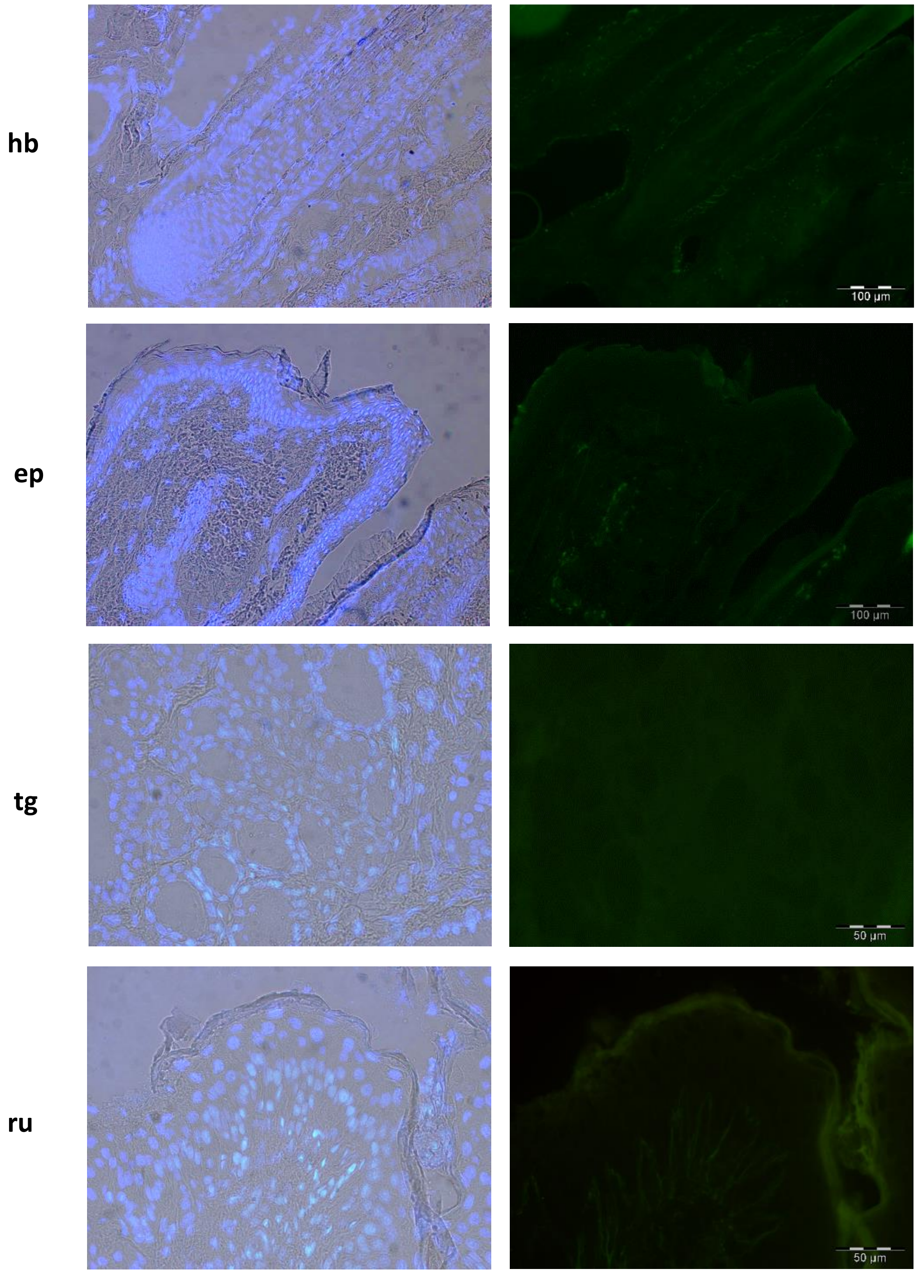

Supplement: Supplementary file 1 [file genes-11-00788-s001.pdf]
